# Epoxidation of Cardanol’s Terminal Double Bond

**DOI:** 10.3390/polym12092104

**Published:** 2020-09-16

**Authors:** Emre Kinaci, Erde Can, John J. La Scala, Giuseppe R. Palmese

**Affiliations:** 1 Department of Chemical and Biological Engineering, Drexel University, Philadelphia, PA 19104, USA; peveenrete@gmail.com; 2 Department of Chemical Engineering, Faculty of Engineering, Yeditepe University, Ataşehir 34755, Istanbul, Turkey; erde.can@yeditepe.edu.tr; 3 Army Research Laboratory, 4600 Deer Creek Loop, Aberdeen Proving Grounds, MD 21005-5096, USA; john.j.lascala.civ@mail.mil

**Keywords:** biobased, epoxy, epoxidation, thermoset, cardanol, reactivity, cure

## Abstract

In this investigation, the terminal double bonds of the side chain epoxidized cardanol glycidyl ether (SCECGE) molecule were further epoxidized in the presence of Oxone^®^ (potassium peroxomonosulfate) and fluorinated acetone. Regular methods for the double bond epoxidation are not effective on the terminal double bonds because of their reduced electronegativity with respect to internal double bonds. The terminal double bond functionality of the SCECGE was epoxidized to nearly 70%, increasing the epoxy functionality of SCECGE from 2.45 to 2.65 epoxies/molecule as measured using proton magnetic nuclear resonance (^1^H-NMR). This modified material—side chain epoxidized cardanol glycidyl ether with terminal epoxies (TE-SCECGE)—was thermally cured with cycloaliphatic curing agent 4-4′-methylenebis(cyclohexanamine) (PACM) at stoichiometry, and the cured polymer properties, such as glass transition temperature (*T*_g_) and tensile modulus, were compared with SCECGE resin cured with PACM. The *T*_g_ of the material was increased from 52 to 69 °C as obtained via a dynamic mechanical analysis (DMA) while the tensile modulus of the material increased from 0.88 to 1.24 GPa as a result of terminal double bond epoxidation. In addition to highlighting the effects of dangling side groups in an epoxy network, this modest increase in *T*_g_ and modulus could be sufficient to significantly expand the potential uses of amine-cured cardanol-based epoxies for fiber reinforced composite applications.

## 1. Introduction

Cardanol, which is the main component of the thermally treated cashew nutshell liquid (CNSL), is characterized by a phenol ring connected to C15 alkyd chains at the meta position that have different degrees of unsaturation [1]. The C15 alkyd chain of the cardanol molecule is found to be completely saturated (5%), mono- (40%), di- (20%) and tri- (35%) unsaturated with the double bonds on the 8–9, 11–12 and 14–15 carbon positions of the side chain [2]. The unique structure of cardanol makes this material a promising candidate for a variety of applications and for many routes of chemical modifications. So far, cardanol has been utilized in many thermosetting applications as vinyl esters [1,3,4], polyols [5,6], polyurethanes [7,8], novolacs [9,10], benzoxazines [11,12,13], epoxies [14,15] and amines [16,17]. Among the different type of thermosetting resins that can be synthesized from cardanol, epoxy resins are an important class of materials because they can be further modified to obtain different materials, such as vinyl ester and polyol resins. Cardanol based epoxies, can be used for protective anticorrosion coatings of metal structures, in adhesive formulations for construction, and as matrices in fiber reinforced composites for marine and automotive applications provided the *T*_g_ is high enough. The direct epoxidation of cardanol can be achieved at the phenolic moiety via the reaction with epichlorohydrin, which leads to phenolic, primary epoxies [15] or through the epoxidation of the side chain unsaturation with peroxyacids or other means, which results in secondary aliphatic epoxies [18].

Epoxidation of the olefins on the cardanol side chain can be achieved via a few different chemistries. The final structure proposed by these studies generally state that the terminal unsaturation is effectively epoxidized. However, their proton nuclear magnetic resonance (^1^H-NMR) and/or epoxy equivalent weight (EEW) titrations do not match with the proposed final structure and suggests that terminal double bonds are not as reactive as internal ones [17,18,19,20]. In a series of studies conducted on the epoxidation of the cardanol side chain in the presence of the m-chloroperoxybenzoic acid catalyst, terminal epoxies were proposed [17,18]. Yet, their EEW, ^1^H-NMR and iodine value results [17,18] do not support the fact that the terminal double bond is epoxidized effectively. In the ^1^H-NMR spectra of the product, poly epoxy cardanol glycidyl ether (PECGE), the multiplet related to the terminal double bonds (5.0 and 5.8 ppm) was clearly observed after the side chain epoxidation reaction. Additionally, the product EEW value 175 g/eq closely matches our pervious study in which the structure of side chain epoxidized cardanol glycidyl ether (SCECGE) was proposed without the terminal epoxies [21]. Furthermore, if the terminal unsaturation were completely epoxidized, there would be 2.80 epoxies per cardanol with an EEW of 155 g/eq based on our structural analysis. The high iodine value (25.8 g/100 g) obtained after the side chain epoxidation reaction [17,18] also indicates that the terminal double bonds are not effectively epoxidized with the current method. Moreover, these terminal epoxies on the cardanol structure were designated as the secondary epoxies on the related ^1^H-NMR spectra [17,18]; however, terminal epoxies have a primary nature and should be recognized as primary epoxies, as primary glycidyl epoxies have significantly higher reactivity than secondary aliphatic [21], and it is likely that primary aliphatic epoxies also have higher reactivity than secondary ones. In another study, the side chain epoxidation of cardanol is achieved via an enzymatic route (lipase) and suggests a molecular structure with terminal epoxies [19,20]. Yet, their ^1^H-NMR analysis also shows that the peaks representing the terminal double bonds (5.0 and 5.8 ppm) were generally unchanged in the spectra [19,20] and thus terminal double bonds are still present in the final structure as per the previous example. The internal unsaturation peaks observed at 5.4 ppm, on the other hand, are significantly reduced in intensity after the synthesis protocol [19,20] suggesting that this enzymatic route is significantly more effective on the internal double bonds than on the terminal ones. In addition, the epoxide content values that are obtained by titrations showed that only 51% of the double bonds were converted to the epoxies [19,20] indicating poor reactivity of the terminal unsaturation sites. In a related study where the side chain of cardanol was epoxidized via hydrogen peroxide and formic acid and was further hydrolyzed to hydroxyl groups [6], the structure of the side chain epoxy cardanol molecule was proposed with terminal epoxies, but again, their ^1^H-NMR analysis clearly showed that most of the terminal unsaturation peaks observed between 5.0 and 5.8 ppm still remained in the molecule’s spectra after the epoxidation reaction, while the peak observed around 5.4 ppm, which represents the internal double bonds, completely disappeared after a 24 h reaction [6]. This again suggests that this common epoxidation method is not effective for the epoxidation of the terminal double bonds. Furthermore, because of the flawed structural analysis of the side chain epoxidized cardanol, the hydrolyzed structure of the cardanol was proposed with terminal hydroxyl groups [6]. Incorrect structural analysis of these molecules may lead to inaccurate stoichiometry calculations and muddled structure–property analysis of resulting polymers. In our previous study it was found that the terminal double bonds are more difficult to epoxidize using common synthetic methods and that more effective means of oxidation are required [21]. 

A recent advance is the use of dioxiranes under room temperature conditions for the epoxidation of olefins [22]. These reagents are generated from Oxone^®^ (a potassium monopersulfate compound) and the parent ketone in the presence of acetonitrile/water/sodium bicarbonate system that allows the accelerating effect of ketones [23,24]. Using fluorinated ketones instead of non-fluorinated ones improves the electronegativity of the formed dioxiranes, and they are significantly more effective for the epoxidation of the less electrophilic terminal double bonds [25,26].

**Figure 1:**
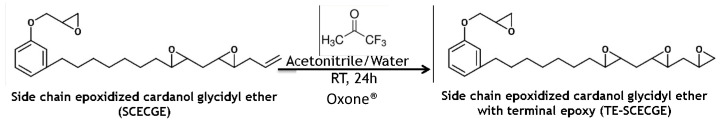
Synthesis scheme of the terminal double bond epoxidation reaction via representative idealized structures.

In this study, the SCECGE molecule with 2.45 epoxies/molecule (1.05 primary epoxy/molecule and 1.40 secondary epoxy/molecule) and 0.32 terminal double bonds/molecule was prepared and further epoxidized at the terminal double bonds of the alkyd chain via Oxone^®^ and fluorinated acetone in the water/acetonitrile/NaHCO_3_ buffer (Figure 1). The epoxidized cardanol was characterized by ^1^H-NMR and epoxy titration and showed that the terminal site was in fact epoxidized. This is of practical importance in designing new materials because it provides a way to eliminate dangling chain ends that are a cause of low *T*_g_ for network polymers derived from cardanol. Thus, epoxy resins were formulated using this material and the extent of cure and thermomechanical properties of the resulting polymers were assessed demonstrating that terminal epoxides improve the cure and polymer properties.

## 2. Materials and Methods 

### 2.1. Materials

Ethyl acetate (>99%), acetonitrile (99.9%), 1,1,1-trifluoroacetone (>98%), sodium chloride (99.9%), sodium bicarbonate (99.9%), magnesium sulfate (>99%) and Oxone^®^ potassium monopersulfate compound (CAS no: 70693-62-8) were purchased from Sigma Aldrich, St. Louis, MO, USA. 4-4′-methylene biscyclohexaneamine PACM (99%) were obtained from Air Products, Allentown, PA, USA. SCECGE, was synthesized as previously reported [21]. All chemicals in this work were used as received.

### 2.2. Epoxidation of the Terminal Double Bond via Oxone^®^

The terminal double bonds presented on the alkyd chain of the SCECGE molecule were epoxidized in the presence of fluorinated acetone and the Oxone^®^ reagent (Figure 1). In a representative procedure, 10 mol (4.0 g) of SCECGE were dissolved in 50 mL 50/50 (*v*/*v*) acetonitrile/water solution and charged into a three-necked round-bottom flask equipped with a reflux condenser and constant magnetic stirring. The contents were allowed to mix at 0 °C for ten minutes and then 4 mL (12.1 equivalent per terminal double bond) 1,1,1 trifluoroacetone was introduced to the reactor and mixed for another thirty minutes at this temperature. Homogenous mixtures of Oxone^®^ 11.9 g (15.1 equivalent per terminal double bond) and sodium bicarbonate (7.01 g, 8.3 equivalent per double bond) in 50/50 *v*/*v* acetonitrile/water were introduced to the reactor at 0 °C in one portion. The contents were allowed to react at room temperature with constant stirring and treated with 50 mL of water after 24 h. The reaction mixture was extracted with ethyl acetate (3 × 50 mL). The combined organic layers were washed with saturated sodium chloride solution (5 × 50 mL), dried over anhydrous magnesium sulfate and rotary evaporated to give the product TE-SCECGE (yield: 30–35%).

### 2.3. Characterization of the TE-SCECGE Resin

Proton magnetic nuclear resonance and epoxy equivalent weight titrations were used to calculate the epoxy functionality of the SCECGE and TE-SCECGE resins and identify and quantify the remaining unsaturation. An ^1^H-NMR (500 MHz, Varian Unity Inova) unit was used with a spectral window of ±2000 Hz, 90° pulse width and 32 scans at 25 °C. EEW titrations were performed in accordance with the ASTM D1652-97 to determine the epoxy content of the resins before and after the epoxidation reaction.

### 2.4. Preparation of Cured Samples of SCECGE and TE-SECCGE with PACM

Polymer samples were prepared by thermally curing SCECGE and TE-SCECGE epoxy resin with the cycloaliphatic curing agent PACM at stoichiometry of epoxides to amine hydrogens. The epoxy–amine combinations were mixed and degassed, cast into the rectangular rubber molds, then thermally cured at 90 °C for 12 h and post-cured at 180 °C for another 3–4 h. The conversion of the epoxy and amine groups was measured by Fourier transform near infrared (FT-NIR) spectrometry using a Nicolet Nexus 670 spectrometer (Thermo Electron Corporation, Waltham, MA, USA), operating in the transmission mode with a deuterated triglycine sulfate (DTGS) detector. FT-NIR spectra were recorded with 32 scans at an 8 cm^−1^ resolution in 4000−8000 cm^−1^ range at room temperature. In addition, the reactivity difference between SCECGE and TE-SCECGE resins towards PACM was determined via DSC (TA Instruments, New Castle, DE, USA, Q2000) by thermally scanning epoxy/amine mixtures from 30 to 180 °C with 2 °C/min heating rate in the nitrogen environment. After the curing was achieved via the first scan, samples were cooled back to −50 °C and reheated to 200 °C at 5 °C/min to obtain the glass transition temperature of the cured material.

### 2.5. Properties of the Cured Epoxy–Amine Polymer

The dynamic mechanical analysis (TA Instruments, Q800) was used to evaluate the cross-link density (*v*), and *T*_g_ of the cured polymers. Rectangular DMA bars with approximate dimensions of 35 mm^3^ × 12 mm^3^ × 3.5 mm^3^ were scanned from −100 °C to well above their glass transition temperature at 1 Hz with a deflection of 15 µm while ramping the temperature at 2 °C/min. The temperature value of the maximum of the loss modulus peak was taken as the *T*_g_ value of the cured polymer. The crosslink density values were calculated using Equation (1), where *E* is the rubbery modulus at a given absolute temperature T, and R is the ideal gas constant [27]. The rubbery plateau storage modulus (E’ at *T*_g_ +50 °C) was used for E in Equation (1).

(1) v=E3RT

Tensile tests were performed on dumb-bell-shaped (type IV) samples of all the fully cured samples of epoxy and amine combinations in accordance with the ASTM D-638 standard test method. Samples were tested at ambient conditions using an extensometer to measure strain with a constant crosshead speed of 1 mm/min and a gauge length of 45 mm. For each formulation, at least 6 tensile specimens were tested and analyzed.

## 3. Results and Discussion

### 3.1. Characterization of the Epoxidized Cardanol

SCECGE was characterized in previous work [21] as summarized in Table 1. The EEW was determined to be 177 g/mol. The results showed that SCECGE had 0.99 aromatic glycidyl groups, 0.06 primary aliphatic epoxy groups and 1.45 secondary epoxy groups with a total of 2.45 epoxy groups per cardanol molecule. SCECGE had 0.32 terminal unsaturation sites remaining per molecule, but no non-terminal unsaturation sites. The average molecular weight of the monomer was 430 g/mol assuming no oligomerization.

**Figure 2:**
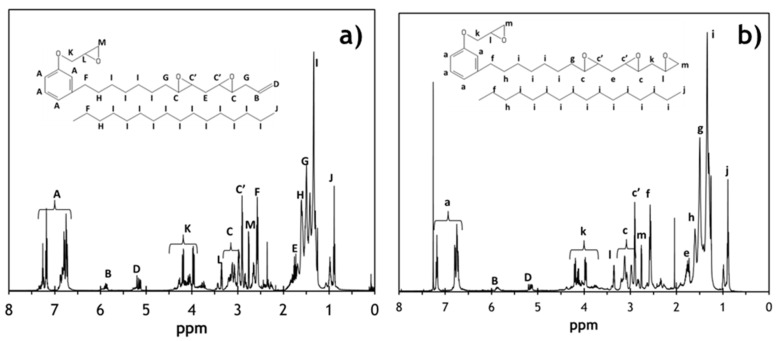
^1^H-NMR analysis of the reactant: (**a**) SCECGE and the product (**b**) TE-SCECGE.

The ^1^H-NMR spectra of the SCECGE and TE-SCECGE are shown in Figure 2a,b, respectively, along with the corresponding peak assignments. In the ^1^H-NMR spectra of the reactant SCECGE, the terminal double bonds are recognized between 5.1 and 5.8 ppm as B and D; and after 24 h of reaction, the intensity of these peaks reduced significantly. Similarly, the peaks representing the primary epoxies, which are designated as k, l and m, slightly increased in intensity in the TE-SCECGE spectra suggesting that a significant amount of terminal double bonds was converted to primary epoxies. The terminal double bond and the primary epoxy functionality of the TE-SCECGE resin were determined via the integration of the related ^1^H-NMR peaks in Figure 2a,b as explained in our previous study [21]. The integral values of the related ^1^H-NMR peaks are also presented in the supplemental information (Tables S1 and S2). The terminal double bond functionality of the SCECGE reduced from 0.32 to 0.10 double bonds/molecule after the epoxidation reaction. Additionally, the primary epoxy functionality of the TE-SCECGE resin was increased by 0.20–1.25 primary epoxy/molecule. The secondary epoxy functionality of the SCECGE resin was calculated as 1.40 epoxies/molecule and did not change after 24 h of reaction. Thus, TE-SCECGE had 2.65 epoxide/molecule where 1.25 was phenolic (0.99) and terminal primary epoxies (0.26), and 1.40 was the secondary, aliphatic epoxy as summarized in Table 1. The 0.02 unsaturation that was not converted to epoxies was lost to side reactions, likely resulting in etherification or alkoxyl formation. This indicates a conversion efficiency of terminal unsaturation to terminal epoxies of 63%. Based on this level of epoxidation, the calculated molecular weight of TE-SCECGE was 437 g/mol assuming no oligomerization.

The EEW of the SCECGE and TE-SCECGE were also determined via epoxy titration and results are presented in Table 1. The EEW of the SCECGE was determined as 177 g/equivalent. The EEW of SCECGE was reduced from 177 to 165 g/equivalent for TE-SCECGE as a result of the addition of primary epoxies. This equated to 2.65 epoxies per TE-SCECGE, an increase of 0.2 epoxies per molecule from SCECGE. This level of epoxidation was in excellent agreement with the NMR analysis. Again, the results confirmed a 63% conversion of the double bonds to primary epoxies and the synthesis protocol did not hydrolyze the existing primary and/or secondary epoxies already present on the SCECGE molecule. When considering the fact that cardanol and cardanol glycidyl ether have 0.38 unsaturation per equivalent, the overall conversion of the terminal unsaturation to primary aliphatic epoxies was 68% after two different means of epoxidation. The calculated molecular weight based on EEW was 437 g/mol, again assuming no oligomerization, which agreed exactly with the ^1^H-NMR analysis.

It is also important to report that we performed the same synthesis protocol using acetone instead of fluorinated acetone and we observed only a very slight epoxidation of the terminal double bond with a conversion around 5–10%. Due to the less electrophilic nature of the terminal double bonds with respect to the inner ones as a result of their primary nature, fluorinated acetone was used along with Oxone^®^ to increase the electronegativity of the oxygen carrier dioxirane formed as a result of the Oxone–ketone reaction [24,25] resulting in the 63% conversion of unsaturation to epoxidation previously discussed. This shows that fluorinated acetone was significantly more effective for the epoxidation of the less electrophilic terminal double bond. 

### 3.2. Curing of the Epoxy Resins with PACM

**Figure 3:**
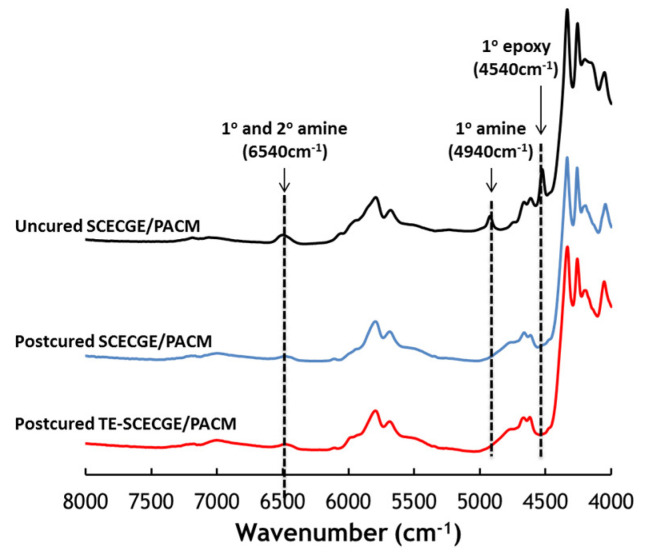
Extent of the epoxy/amine curing reaction via near-IR.

Figure 3 shows the near-IR spectra of SCECGE and TE-SCECGE samples with PACM after post cure along with the representative uncured epoxy/amine spectra. The primary epoxy and primary amine peaks observed at 4540 and 4940 cm^−1^, respectively, completely disappeared while the peak representing both the primary and secondary amine at 6570 cm^−1^ was still observed as a slight hump after the post-cure step in both SCECCGE and TE-SCECGE spectra despite the stoichiometric amount of amine [28]. The reason for the incomplete curing for the SCECGE and TE-SCECGE resins can be explained by the less reactive nature of the secondary epoxides, which cannot be cross-linked to a high degree as we demonstrated previously [21]. However, the overall extent of the cure for the TE-SCECGE, which was calculated around 84%, was slightly higher than the SCECGE resin (82%) as a result of the extra primary epoxy, which can completely react with amines and contribute to network formation.

**Figure 4:**
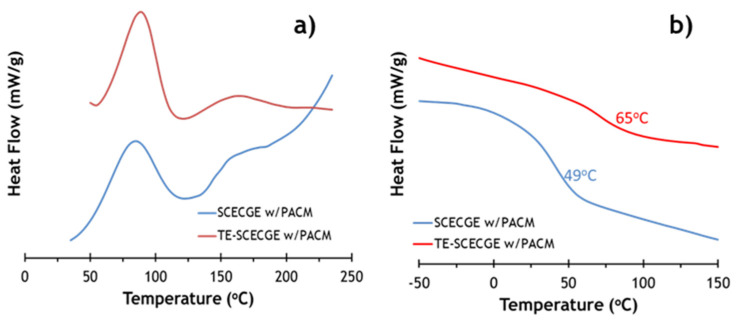
(**a**) First run DSC showing exothermic reaction peaks associated with primary (lower temperature) and secondary epoxy reaction of SCEGE and TE-SCEGE cured with PACM and (**b**) the second DSC runs of the corresponding cured epoxy/amine mixtures showing distinct *T*_g_ transitions.

DSC was employed to confirm the extent of curing of both epoxy resins and to compare the reactivity difference between SCECGE and TE-SCECGE in the presence of the cycloaliphatic curing agent PACM. DSC scans of uncured SCECGE and TE-SCECGE resin prepared with PACM at stoichiometry are presented in Figure 4. In the first heating cycle where epoxy and amine curing reaction occurred, the temperature associated with the maximum in the heat release rate of the curing reactions (*T*_max_) and the heat of the curing reaction (Δ*H*_cure_) values were determined and presented in Table 2. Figure 4 shows that SCECGE and TE-SCECGE epoxy resins, which possessed both primary and secondary epoxy functionality, had a two-step curing process. The peak at lower temperatures, observed around 70–80 °C, was assigned to the primary epoxy curing reaction and the second peak, observed as a slight hump around 120–125 °C, was assigned to the secondary epoxy curing reaction [21]. Δ*H*_cure_ values—obtained via the area under two exotherms presented in Table 2—showed the Δ*H*_cure_ value of the SCECGE was around 45 kJ/mol of epoxy, and after the addition of the terminal primary epoxy, the Δ*H*_cure_ value of TE-SCECGE increased to 52 kJ/mol of the epoxy. Previous work [21] showed that the Δ*H*_cure_ value of the secondary epoxies is extremely low (8 kJ/mol of epoxy) and this significant increase can be associated with the presence of extra primary epoxies on the side chain. This supports our claim that we have added terminal epoxies to the side chain of cardanol glycidyl ether. More so, it shows that primary aliphatic epoxy groups have similar reactivity to glycidyl groups and are far more reactive than secondary epoxy groups.

Our previous analysis also showed that the Δ*H*_cure_ values for DGEBA based epoxies are 90 kJ/mol (45 kJ/mol epoxy), which correspond to the heat of reaction value of a primary epoxy and amine found in the literature [29,30]. Given the increase of 0.2 primary epoxies and the same heat of reaction for primary aliphatic epoxies vs. glycidyl epoxies, the predicted Δ*H*_cure_ should be 56 kJ/mol, which is higher than the 52 kJ/mol measured. To obtain this value the heat of reaction would have to be 29 kJ/mol for primary aliphatic epoxies. This indicates that the reaction of primary aliphatic epoxies is less exothermic, and these epoxy groups are less reactive than glycidyl groups but are still far more reactive than secondary epoxy groups (8 kJ/mol). However, TE-SCECGE resin gels very quickly and a significant amount of polymerization may have occurred during sample preparation for DSC measurements; thus, the calculated value of 29 kJ/mol for terminal primary epoxy was most likely lower than the actual value of Δ*H*_cure_. 

### 3.3. Comparison of the Thermomechanical and Mechanical Properties of SCECGE and TE-SCECGE

DSC scans of the second heating cycle (Figure 4b) had a slope change attributed to the materials glass transition temperature (Table 2). The glass transition temperatures of the SCECGE and TE-SCECGE cured with PACM were measured as 49 and 65 °C, respectively, via DSC. This shows that the addition of only 0.2 reactive primary terminal epoxies resulted in a relatively large 16 °C in the glass transition of this material. More so, it again corroborated the fact that special reaction conditions are necessary to produce terminal epoxies on the alkyl chain of cardanol.

**Figure 5:**
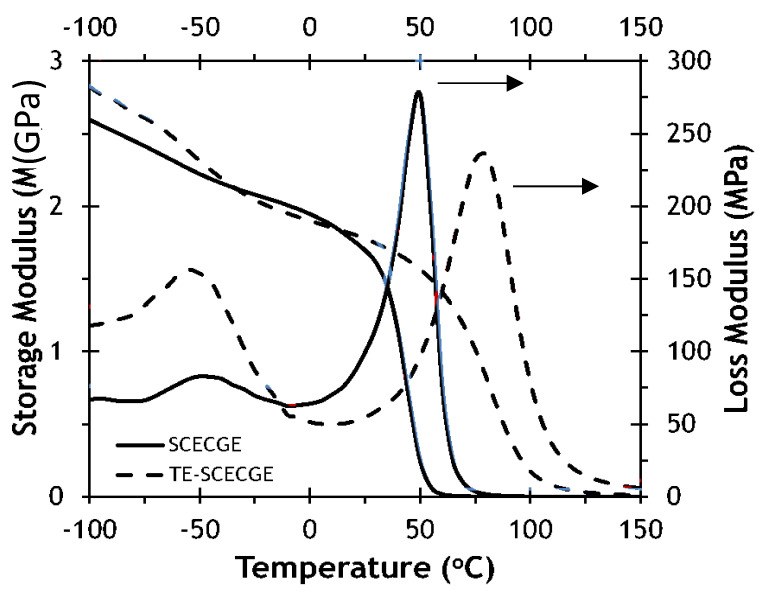
Temperature dependence of the storage and loss modulus of SCECGE and TE-SCECGE cured with PACM at stoichiometry.

Figure 5 shows the temperature dependence of the storage modulus and loss modulus curves for SCECGE and TE-SCECGE cured with PACM at stoichiometry. The cross-link density of the epoxy/amine formulations was determined via DMA as previously explained and presented in Table 2. The cross-link density value was determined as 375 mol/m^3^ for the SCECGE, and after the addition of the terminal epoxy, the cross-link density of the cured polymer increased to 625 mol/m^3^ because of the presence of an extra reacting site. We also calculated the cross-link density of these polymers by using a simple method proposed by Hill et al. for partial network formation to compare the cross-link density measurements [31]. The cross-link density of the SCECGE and TE-SCECGE epoxy was estimated as 745 mol/m^3^ and 1100 mol/m^3^ via the Hill’s method. The values were higher for the Hill’s method; this is typical because this theoretical method does not factor in intramolecular cyclization. Yet, the Hill’s method shows the same trend and magnitude of increase in the cross-link density as we go from SCECGE to TE-SCECGE. The addition of the terminal epoxy to the side chain increased the epoxy functionality of the TE-SCECGE resin, and thus a higher cross-link density value was observed for TE-SCECGE. In addition, the glass transition temperature of the fully cured epoxy/amine mixture increased from 52 to 69 °C as determined via DMA as a result of the additional cured epoxy groups. These *T*_g_ values were very similar, albeit slightly higher, to that measured by DSC, and the *T*_g_ increment of 17 °C measured by DMA was nearly identical to that measured via DSC. In addition, the β transition that is observed at lower temperatures, which is associated with the rotation of groups along the polymer backbone, had increased intensity for the TE-SCECGE with respect to SCECGE. DGEBA-PACM samples also have pronounced β transitions. TE-SCECGE is more similar to DGEBA in that most of the molecule end groups are tied into the network, whereas there are large amounts of dangling chain ends in SCECGE. Tying these dangling chain ends into the network in TE-SCECGE constrains significant movement of the chain at temperatures below the *T*_g_ but may then enable some of the lower energy transitions, such as molecular rotations [32].

The mechanical properties such as the Young’s modulus (*E*), tensile strength (*σ*) and tensile strain (*ε*) of the fully cured epoxy/amine formulations were evaluated at room temperature and presented in Table 3. The Young’s modulus of the SCECGE and TE-SCECGE was determined as 0.88 GPa and 1.3 GPa, respectively. Similarly, the tensile strength of the epoxy/amine formulations increased from 13 to 20 MPa, which also supports the fact that the addition of the terminal epoxy improved the mechanical properties of the epoxy/amine network significantly. The reason for the improvement in the mechanical properties can be explained by the increased rigidity of the cured network as a result of increased cross-link density after the addition of the terminal epoxies. Interestingly, the failure strain values obtained for the TE-SCECGE did not show a significant improvement with respect to SCECGE. An increment from 3.1% to 4.0% was observed after the terminal double bond epoxidation. However, as polymers are stiffened and cross-link density is increased, elongation to failure generally decreases. The fact that it increased indicates that TE-SCECGE was acting more effectively as a chain extender in the epoxy-amine resins system as a result of more epoxies per molecule and more reactive epoxies per molecule. SCEGCE likely has more terminal cardanol units because there was only one reacted epoxy group per molecule for a significant percentage of the polymer.

The combinatorial analysis as done in La Scala et al. for triglyceride based thermosets was also performed on cardanol [33,34]. Using the distribution of reactive sites before reaction, F(*N*), the distribution of functional groups after reaction can be determined using a binomial distribution [33,34]. The probability of having *n* functional groups on the cardanol alkyl chain with *N* reactive sites was calculated using Equation (2) [34].

(1) $$P\left(N,n,\xi \right)=C\left(N,n\right)\xi^{n}{\left(1-\xi \right)}^{N-n}F\left(N\right)$$

where *ξ* is the extent of reaction and *C* (*N*, *n*) is the number of different ways the *n* functional groups can be arranged on the cardanol alkyl chain with N reactive sites (i.e., *C* (*N*, *n*) is the combinatorial function or binomial coefficient). The percentage of *n*-functional cardanol chains, *p* (*n*,*ξ*), is:

(2) $$p\left(n,\xi \right)=\underset{N}{\sum }P\left(N,n,\xi \right)$$

This methodology assumes equal reactivity of each functional group with some exceptions. Past work on SCECGE showed that 0.06 of 0.38 unsaturation were converted to primary epoxies, and the extent of epoxidation of the secondary epoxies was 0.91 [21]. Regarding the cured species, the primary glycidyl and alkyl epoxy functionality was shown to react to nearly 100%, while the secondary epoxy functionality only cured to 60% [21]. Clearly, all saturated alkyl chains remained unfunctionalized and uncured. Using this combinatorial function, we could calculate the probability of having each species and the overall percentage of 0–3 epoxy and cured epoxy cardanol alkyl chains. Factoring in the phenyl glycidyl group, we had functionality ranging from 0 to 4, as listed in Table 4. The results show that in SCECGE, 33.0% of the molecules had fewer than 2 cured epoxies and thus would result in network defects. This amount was reduced by 3.2% in TE-SCEGCE down to 29.8% after the addition of the terminal epoxy because addition of the terminal epoxy resulted in a better extent of cure for the TE-SCECGE polymer. Thus, this network would likely have better strength and modulus and elongation to failure. However, relative to other cardanol networks—like NC514 that has nearly two cured epoxies per monomer and nearly 0% of cardanol units with fewer than two uncured epoxies—TE-SCECGE clearly had far higher amounts of such species resulting in lower elongation to break, where elongation to break in NC514-PACM was 32.7 ± 2.6 [21]. 

## 4. Conclusions

The terminal double bond of the cardanol molecule was epoxidized at 63% conversion in the presence of Oxone^®^ and the fluorinated acetone complex. Due to their primary nature and low electronegativity with respect to inner double bonds, common synthetic methods were not able to effectively epoxidize the terminal double bonds. The epoxy functionality of the SCECGE molecule with 2.45 epoxies/molecule increased to 2.65 epoxy/molecule after the epoxidation reaction with Oxone^®^ and the fluorinated acetone complex. Interestingly, this small level of increased epoxidation resulted in relatively large improvements in the polymer’s properties. The glass transition temperature of the cured material was increased by about 16 °C and the Young’s modulus increased from 0.88 to 1.3 GPa. These relatively large property increases were a result of the much higher reactivity of the terminal primary aliphatic epoxies relative to the secondary aliphatic epoxies. 

## Figures and Tables

**Table 1:** Epoxy content of the side chain epoxidized cardanol glycidyl ether (SCECGE) and TE-SCEGE resins as obtained via ^1^H-NMR and epoxy equivalent weight (EEW) titrations.

Epoxy Resin	Terminal Double Bond Functionality (per Molecule)	Primary Epoxy Functionality (per Molecule)	Secondary Epoxy Functionality (per Molecule)	Total Epoxy Functionality (per Molecule)	EEW (g/Equivalent)
SCECGE [21]	0.32	1.05	1.40	2.45	177
TE-SCECGE	0.10	1.25	1.40	2.65	165

**Table 2:** Thermomechanical property comparison of SCECGE and TE-SCECGE.

Epoxy Resin	Δ*H*_RXN_ (J/mol)	*T*_g_ (DSC) (°C)	Cross-Link Density (mol/m^3^)	*T*_g_ (DMA) (°C)
SCECGE [21]	45	49	375	52
TE-SCECGE	52	65	625	69

**Table 3:** Comparison of the mechanical properties as obtained via tensile tests.

Epoxy Resin	Young’s Modulus (*E*) (GPa)	Tensile Strength (*σ*) (MPa)	Failure Strain (*ε*) (%)
SCECGE [21]	0.88 ± 0.1	13 ± 2	3.1 ± 0.4
TE-SCECGE	1.24 ± 0.3	24 ± 7	4.0 ± 1.2

**Table 4:** Calculated distribution of epoxies and cured epoxies on the SCECGE and TE-SCECGE.

Functionality	SCECGE Epoxies	TE-SCECGE Epoxies	SCECGE Cured Epoxies	TE-SCECGE Cured Epoxies
0	0	0	0	0
1	8.4	8.3	33.0	29.8
2	44.5	41.7	47.9	42.8
3	42.5	30.4	17.5	20.4
4	4.5	19.6	1.6	7.1

## Supplementary Materials

The following are available online at https://www.mdpi.com/2073-4360/12/9/2104/s1, Equation (S1): Terminal double bond functionality, Equation (S2): Secondary epoxy functionality, Equation (S3): Primary epoxy functionality, Table S1: Normalized peak intensity values as obtained via ^1^H-NMR traces of the reactant SCECGE, Table S2: Normalized peak intensity values as obtained via ^1^H-NMR traces of the product TE-SCECGE.
