# Supplementary material for: Epoxidation of Cardanol’s Terminal Double Bond
Source: Polymers (Basel). 2020 Sep 16;12(9):2104. doi: 10.3390/polym12092104 (PMC7570343; doi:10.3390/polym12092104)
Supplement: Supplementary file 1 [file polymers-12-02104-s001.pdf]

Epoxidation of Cardanol's Terminal Double Bond

Emre Kinaci [1], Erde Can [2], John J. La Scala [3] and Giuseppe R. Palmese [1,*]

[1] Department of Chemical and Biological Engineering, Drexel University, Philadelphia, PA 19104, USA; peveenrete@gmail.com
[2] Department of Chemical Engineering, Faculty of Engineering, Yeditepe University, Ataşehir 34755, Istanbul, Turkey; erde.can@yeditepe.edu.tr
[3] Army Research Laboratory, 4600 Deer Creek Loop, Aberdeen Proving Grounds, MD 21005-5096, USA; john.j.lascala.civ@mail.mil
* Correspondence: grp27@drexel.edu; Tel.: +1-215-895-5814

Received: 29 August 2020; Accepted: 14 September 2020; Published: date

Supplementary Information

^{1}H-NMR analysis of SCECGE and TE-SCECGE resins.

Equations S1, S2, S3: Equations to calculate different monomer functionalities for SCECGE and TE-SCECGE resins.

Terminal double bond functionality: $$((I_B + I_D)/I_J) \times (3/3) \quad \text{(S1)}$$

Secondary epoxy functionality: $$(I_C)/I_J) \times (3/1) \quad \text{(S2)}$$

Primary epoxy functionality: $$(I_L)/I_J) \times (3/1) \quad \text{(S3)}$$

Table S1. Normalized peak intensity values as obtained via ^{1}H-NMR traces of the reactant SCECGE.

Peak Designation	Shift (ppm)	Peak Name	Normalized Intensity
B–D	5.2–5.8	Terminal double bond (-C**H**2=C**H**)	0.96 (3H)
C′	2.9–3.2	Secondary epoxy (CH-O-C**H**)	1.40 (1H)
L	3.4	Primary epoxy & terminal epoxy CH_2–C**H**-O-CH_2	1.05 (1H)
J	0.88	Terminal methyl (-C**H**3)	3 (3H)

Table S2. Normalized peak intensity values as obtained via ^{1}H-NMR traces of the product TE-SCECGE.

Peak Designation	Shift (ppm)	Peak Name	Normalized Intensity
B–D	5.2–5.8	Terminal double bond (-C**H**2=C**H**)	0.30 (3H)
C′	2.9–3.2	Secondary epoxy (CH-O-C**H**)	1.40 (1H)
L	3.4	Primary epoxy & terminal epoxy CH_2–C**H**-O-CH_2	1.25 (1H)
J	0.88	Terminal methyl (-C**H**3)	3 (3H)

© 2020 by the authors. Licensee MDPI, Basel, Switzerland. This article is an open access article distributed under the terms and conditions of the Creative Commons Attribution (CC BY) license (http://creativecommons.org/licenses/by/4.0/).
